# A Case of Budd-Chiari Syndrome Secondary to Tumor Thrombosis

**DOI:** 10.7759/cureus.55330

**Published:** 2024-03-01

**Authors:** Nirmay Sonar, Zaynah Sadiq, Gurvinder Kaur, Shohan Pervaze, Nicholas Cook

**Affiliations:** 1 Internal Medicine, Norton Community Hospital, Norton, USA; 2 Internal Medicine, Ballad Health, Norton, USA; 3 Hematology and Oncology, Norton Community Hospital, Norton, USA

**Keywords:** cancer, inferior vena cava tumor thrombosis, tumor thrombosis, budd-chiari syndrome (bcs), bcs, budd-chiari syndrome

## Abstract

Budd-Chiari syndrome (BCS) is a rare constellation of conditions due to obstruction of venous flow from anatomical levels ranging from the hepatic veins to the confluence of the inferior vena cava (IVC) and right atrium. The resulting retrograde flow of blood leads to hepatomegaly, ascites, and liver failure among other features. Our case highlights the clinical features, diagnostic challenges, and management of a patient with a tumor thrombus from a metastatic prostate adenocarcinoma in a 67-year-old male leading to BCS. This patient, with a past history of prostate adenocarcinoma and aortic valve replacement on chronic warfarin anticoagulation, presented with acutely worsening abdominal pain and a distended abdomen, and imaging revealed an IVC filling defect. Subsequent imaging with a piflufolastat prostate-specific PET showing increased uptake in the IVC elucidated the diagnosis of tumor thrombosis.

Management considerations include aggressive therapy and optimization of quality of life. The patient was offered both options, and options including surgical shunting, bypasses, and anticoagulation were discussed. After shared decision-making, the patient and family opted to choose the pathway of palliative radiation and anticoagulation.

## Introduction

Budd-Chiari syndrome (BCS) is due to the obstruction of the venous flow from the hepatic venous system [1]. This anatomical level of obstruction can vary, from the inferior vena cava (IVC) to the right atrium. The prevalence of BCS in Western countries is estimated at 1 in 4 million [2]. Hypercoagulable states are the commonest etiologies, and a highlight on myeloproliferative disorders associated with JAK2 is made, other causes are extraluminal or intraluminal compression of vessels by tumors, trauma, and IVC webs [3]. Other notable causes include dacarbazine, inflammatory bowel disease, etc. BCS may be idiopathic in some cases [3].

As there is obstruction, the retrograde flow causes hepatic congestion and tissue swelling and can eventually lead to hepatic injury and tissue necrosis [4]. Increased portal pressure leads to extravasation of fluid into the peritoneal space, splenic congestion, and decreased renal perfusion [4].

Patients often present with features such as abdominal pain, likely due to a multitude of causes, one of them being hepatomegaly causing stretching of the liver capsule, ascites, and splenomegaly [5]. Patients can also present with jaundice, and as there is more hepatic dysfunction, patients may have hepatic encephalopathy [5].

## Case presentation

**Figure 1:**
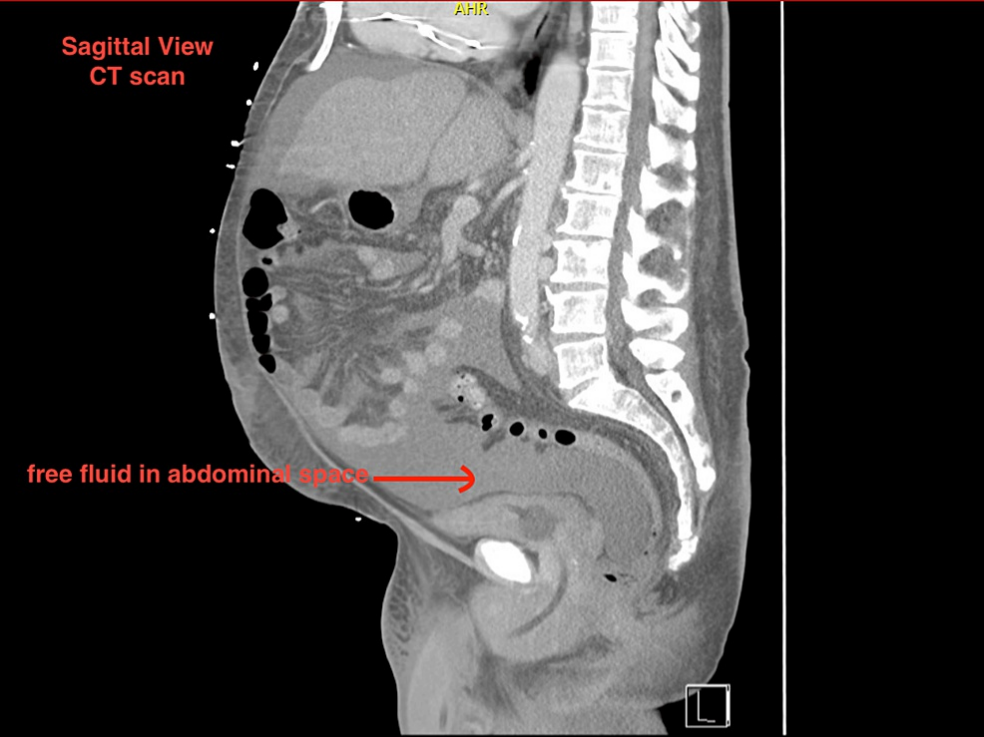
Sagittal view of abdominal CT scan The red arrow indicates free fluid in abdominal fluid. IVC thrombus is not visualized in this view, refer to Figure 2 for an additional view. IVC: Inferior vena cava

A 67-year-old male with a past medical history of prostate adenocarcinoma and aortic valve replacement on warfarin anticoagulation presented to us with complaints of abdominal pain. The patient had chronic abdominal pain and myalgias for years, but this pain was different in character, he described this pain as low intensity, dull in character over the epigastric region, and worsening over the last couple of days, with no relief to his home pain medication (acetaminophen-oxycodone) for the last several hours, associated with bloating and reached a point where he could not lie down, which warranted him to come to the emergency department. The patient's vitals were significant for a temperature of 98.8°F, blood pressure of 89/58 mmHg, pulse of 102 beats per minute, respiratory rate of 16 breaths per minute, saturating 96% on room air. On the evaluation of the patient, he was in acute distress and had a distended and tense abdomen. Imaging with a contrast-enhanced CT scan (intravenous contrast) of the abdomen revealed massive ascites, hepatomegaly with possible cirrhosis, an irregular filling defect in the IVC suggestive of IVC thrombus, and lumbar osteopenia. Additional labs at presentation as detailed in Table 1 are significant for an elevated alkaline phosphatase (ALP), normal liver function, hematuria, and urinary tract infection. Figure 1 shows the ascites and enlarged liver size.

**Table 1:** Significant laboratory values from the initial presentation in the ED Reference values from standardized laboratory reference range. BUN: Blood urea nitrogen; g: Grams; dL: Deciliter; K: Count in thousands; uL: Microliter; mmoL: Millimoles; L: Liter; mg: Milligrams; U: Units; hpf: High powered field

Test	Observed lab value	Normal reference range
Hemoglobin (in g/dL)	10.1	13.9-16.8
Leukocyte count (in K/uL)	7.6	3.5-11.0
Platelet count (in K/uL)	191	150-400
Sodium (in mmoL/L)	133	135-145
Potassium (in mmoL/L)	3.8	3.5-5.0
Creatinine (mg/dL)	1.1	0.5-1.35
BUN: Creatinine ratio	19	10-20
Aspartate transaminase (AST) (in U/L)	39	0-37
Alanine transaminase (ALT) (in U/L)	13	0-53
Alkaline phosphatase (ALP) (in U/L)	303	39-117
Total bilirubin (in mg/dL)	1.4	0.3-1.2
Albumin (in g/dL)	3.5	3.5-5.2
Lactate (in mmoL/L)	3.1	0.7-2.0
Lipase (in U/L)	32	23-300
International normalized ratio (INR)	2.6	1.5-2.5
Urine appearance	Bloody	Pale yellow
Leukocyte esterase	Positive	Negative
Urine leukocytes (in count/hpf)	5-9	0-4
Urine bacteria	Positive	Negative
Urine red blood cells (in count/hpf)	25-49	0-4

With the finding of the IVC thrombus on a chronically anticoagulated patient, vascular surgery was consulted for the recommendation, and based on the patient's record, his warfarin target was 2-3.5, which ruled out failure of anticoagulation, and their recommendation was to continue warfarin therapy (home dose of 6 mg) with INR monitoring. The patient was admitted to the observation unit for symptomatic ascites. UTI treatment with ceftriaxone was given. Fluid restriction, supportive treatment, and general surgery were consulted for diagnostic paracentesis, which showed chemistry consistent with a transudative fluid, sterile, and without any white cells. The patient subsequently clinically improved the next day and was discharged from the hospital to return to his follow-up with the oncology office next week for his cancer treatments and vascular surgery.

**Figure 2:**
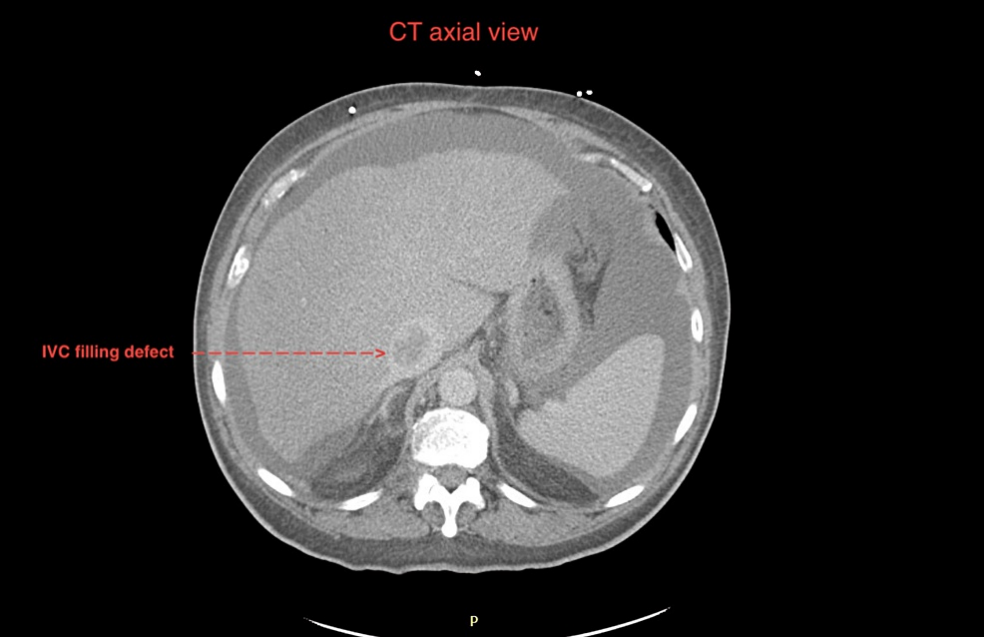
Axial view on CT scan with filling defect in IVC (with red arrow) IVC: Inferior vena cava

**Figure 3:**
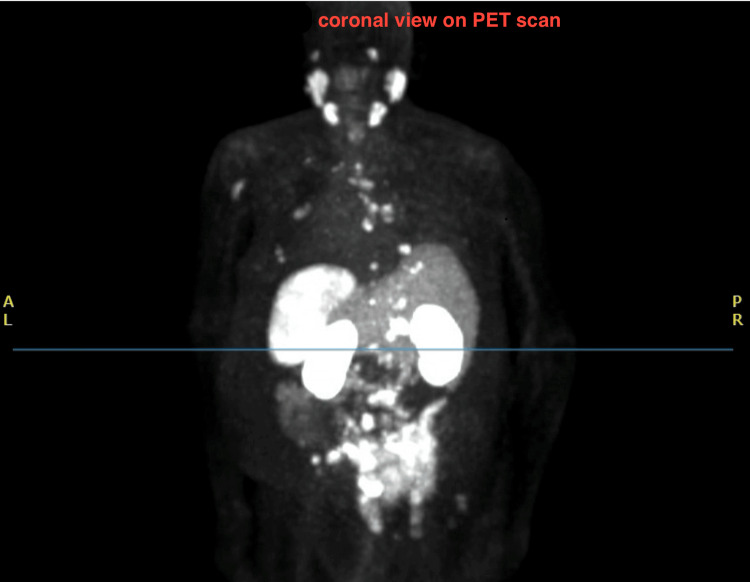
PET piflufolastat F-18 scan in coronal view showing uptake in multiple areas Piflufolastat F-18 PET scan is an FDA-approved PET scan against prostate-specific membrane antigen (PSMA)

**Figure 4:**
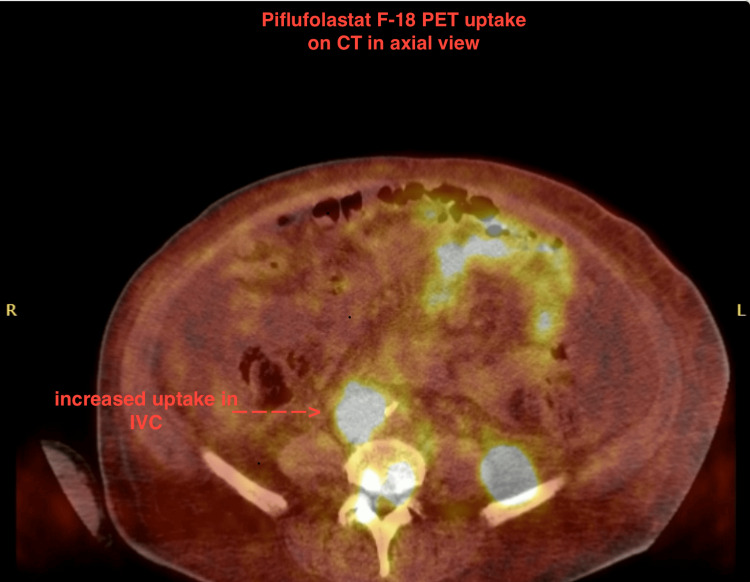
Interposed piflufolastat F-18 PET uptake on CT scan in axial view showing increased uptake in IVC (red arrow) IVC: Inferior vena cava

In regard to his prostate cancer, the patient was staged as stage IVB (grade group 5, prostate-specific antigen (PSA) 37.9) and he was started on Leuprorelin (Eligard) and abiraterone. He was then scheduled for a piflufolastat (Pylarify) PET scan (against prostate-specific membrane antigen (PSMA)), based on the ALP and bone findings of the CT. The PET-CT scan showed increased uptake in the left humerus, left 4th rib, right 5th rib, L4-L5, sacrum, and right femur suggestive of bone metastasis and increased uptake in the IVC, which suggested tumor thrombosis. Figure 2 highlights the IVC filling defect as seen on an axial cut. The increased uptake in multiple tissues is seen in Figure 3. Figure 4 shows increased uptake of the piflufolastat in the IVC suggestive of a tumor thrombus. The patient had a Rotterdam score of 1.8626, which put him in class 3. The plan outlined from this point onward was to start palliative radiation therapy and evaluation of an IVC stent. The patient did receive 3000 centiGray (cGy) radiation over 10 fractions to the L2 sacrum region with the radiation oncology team.

After an extensive review, shared decision-making, and evaluation of risks and benefits, the patient then decided to focus on the quality of life treatments and chose palliative radiation but not a stent placement, he was given adequate pain control and was established with the palliative care team.

## Discussion

BCS, also described as obliterating endophlebitis of the hepatic veins described by British internist George Budd and Hans Chiari, an Austrian pathologist in 1899 is a condition of obstruction of hepatic venous outflow. Worldwide prevalence is around 1 in 100,000 while the disease is rarer in Western countries [2,5]. BCS can be described as a rare constellation of conditions due to obstruction of venous flow at the anatomical levels from the IVC to the right atrium [1].

The major causes of BCS are typically hypercoagulable states, in which myeloproliferative disorders account for about 40-50% and other causes such as factor V Leiden, antiphospholipid syndrome, and paroxysmal nocturnal hemoglobinuria account for another 20% of cases [3]. These are typically classified as primary BCS and the remaining fraction of cases, caused by mechanical causes, such as extrinsic and intrinsic compression of vessels such as tumors, mechanical obstruction from trauma, and adhesions contribute to some number of cases [6].

Clinical features arise due to the retrograde flood of blood through the hepatic portal system. These include but are not limited to abdominal pain, hepatomegaly, abdominal distention which can be a late feature and secondary to ascitic fluid collection in the peritoneal space, nausea, vomiting, and jaundice [5]. In severe cases, patients may present with hepatocellular failure, which may present as encephalopathy and an alteration in mentation [7].

A thorough history and physical examination are imperative in working up these patients. History of cancer, type of cancer, and stage may help in understanding the etiology and overall plan for the specific patient. A review of oncology records may also increase the clinical suspicion of BCS secondary to tumor thrombosis. Classically, a sudden onset of abdominal pain and ascites, in a background of relatively preserved hepatic function may be considered due to BCS [3,5].

Additional workup with hematologic investigations, blood counts, chemistry, and coagulation profile are supplemental to diagnosis. Ruling out coagulation disorders is necessary for the diagnosis of BCS secondary to tumor thrombosis [8]. JAK2 mutation testing for myeloproliferative disorders can assist in ruling those out [9]. Imaging with modalities such as CT/MRI with vascular contrast is typically preferred for BCS [10]; however, they may reveal filling defects and not the nature of the tissue causing obstruction. A PET scan will show uptake consistent with the tumor tissue which can guide the diagnosis. In this case, note that the patient was on a therapeutic level of anticoagulation, which additionally ruled out a cause of anticoagulation failure. Additionally, the PET scan performed was a piflufolastat scan, specific to prostate-specific membrane antigen [11]. Figures 2, 3, and 4 impress upon the images seen, highlighting the filling defect in the IVC and increased uptake on the piflufolastat PET scan. The gold standard for diagnosing this would logically be an extraction biopsy performed intravascularly to identify the histochemical characteristics of the tissue including sinusoidal disease [5]; however, in resource-constrained environments, this may prove challenging. Additionally, this intervention requires a risk and benefit discussion among the treatment team and the patient’s goals of care.

Management for a tumor-induced obstruction can be divided into interventional and non-interventional sections. Important for any clinician to assess the goals of care, the quality of life, and treatment goals for a patient. In the general approach to BCS from any etiology interventional procedures such as shunting, dilation, and portocaval shunting can be considered [12]. After the removal of the thrombus and restoration of flow, patients typically do not gain significant benefits due to the risks of procedures [13-15]. Following this, patients would require treatment of their primary pathology. Hypercoagulable states can be treated with anticoagulation [1]. Malignancy and tumor thrombosis from a prostate adenocarcinoma as described in this case require a plan that outlines multidisciplinary support including oncology, palliative care, radiation oncology, and urology. Early involvement in palliative care to understand the goals of treatment with the patient and family led to more comfort-oriented care and optimization of the quality of life in this case.

A review of long-term prognosis with patients with BCS showed that patients with interventions such as anticoagulation, thrombolysis, shunting, and liver transplant stepwise provide good survival; however, most of the deaths occurred in the first two years of diagnosis [14]. Literature is limited on the prognosis secondary to tumor thrombosis; however, there are reports of rapid progression to liver failure and poor prognosis [15]. The Rotterdam score can be used for the prediction of intervention-free survival, which incorporates clinical and laboratory values and predicts the prognosis of patients [15].

## Conclusions

This case reflects light on BCS secondary to tumor thrombosis from metastatic prostate adenocarcinoma and highlights the complexity of diagnosis and management, including troubleshooting in these patients. The fact the patient was on warfarin chronic anticoagulation, the clinical presentation of abdominal pain with ascites, and imaging findings with filling defects make the consideration of BCS in this patient. Additionally, the uptake seen in the IVC on piflufolastat PET scan to PSMA elucidates the etiology. Additional mentions in terms of a multidisciplinary approach to management are made, with the integration of specialties and prioritizing patient preferences. Literature on prognosis, especially secondary to tumor thrombosis is limited, however reports suggest they follow an aggressive course and poor prognosis. It is important to consider the aggressive and comfort-oriented measures in these patients. In this case, the patient was offered modalities such as shunting, and after shared decision-making, the patient opted to choose the comfort-oriented pathway.
